# Age-Related Skeletal Dynamics and Decrease in Bone Strength in DNA Repair Deficient Male Trichothiodystrophy Mice

**DOI:** 10.1371/journal.pone.0035246

**Published:** 2012-04-10

**Authors:** Claudia Nicolaije, Karin E. M. Diderich, S. M. Botter, Matthias Priemel, Jan H. Waarsing, Judd S. Day, Renata M. C. Brandt, Arndt F. Schilling, Harrie Weinans, Bram C. Van der Eerden, Gijsbertus T. J. van der Horst, Jan H. J. Hoeijmakers, Johannes P. T. M. van Leeuwen

**Affiliations:** 1 Department of Internal Medicine, Erasmus MC, Rotterdam, The Netherlands; 2 MGC Department of Cell Biology & Genetics, Center for Biomedical Genetics, Erasmus MC, Rotterdam, The Netherlands; 3 Department of Trauma, Hand, and Reconstructive Surgery, School of Medicine, Hamburg University, Hamburg, Germany; 4 Department of Orthopedics, Erasmus MC, Rotterdam, The Netherlands; Tulane University Health Sciences Center, United States of America

## Abstract

Accumulation of DNA damage caused by oxidative stress is thought to be one of the main contributors of human tissue aging. Trichothiodystrophy (TTD) mice have a mutation in the *Ercc2* DNA repair gene, resulting in accumulation of DNA damage and several features of segmental accelerated aging. We used male TTD mice to study the impact of DNA repair on bone metabolism with age. Analysis of bone parameters, measured by micro-computed tomography, displayed an earlier decrease in trabecular and cortical bone as well as a loss of periosteal apposition and a reduction in bone strength in TTD mice with age compared to wild type mice. *Ex vivo* analysis of bone marrow differentiation potential showed an accelerated reduction in the number of osteogenic and osteoprogenitor cells with unaltered differentiation capacity. Adipocyte differentiation was normal. Early in life, osteoclast number tended to be increased while at 78 weeks it was significantly lower in TTD mice. Our findings reveal the importance of genome stability and proper DNA repair for skeletal homeostasis with age and support the idea that accumulation of damage interferes with normal skeletal maintenance, causing reduction in the number of osteoblast precursors that are required for normal bone remodeling leading to a loss of bone structure and strength.

## Introduction

Osteoporosis, caused by a natural loss of estrogens, has been the main focus of the bone field for many years. However, recently a growing body of evidence supports a more general, age-related form of bone loss, which occurs independent of changes in sex steroid hormone levels and starts at an early age [1]. Epidemiological studies pin-point the onset of this age-related bone loss right after obtaining peek bone mass in humans [2], [3]. Additionally, early onset and gradual bone loss has also been found in several mouse strains independent of a sudden loss of estrogens starting 2–3 months after birth [4], [5]. This age-related bone loss is characterised by a slower, more gradual decline in bone mass than in case of post menopausal bone loss [6].

Oxidative stress has been postulated as one of the contributing mechanisms behind age-related bone loss [1]. Oxidative stress is induced by reactive oxygen species (ROS) formed as by-products of mitochondrial aerobic metabolism and fatty acid oxidation [7]. ROS at basal levels play an important role in a broad spectrum of signalling pathways [8]. An increase in ROS production in response to external stimuli can induce oxidative stress which causes DNA and protein damage that can lead to apoptosis [9]. All cells have a battery of defence mechanisms to prevent ROS-induced damage. Anti-oxidant enzymes scavenge ROS and reduce them to an inactive state. Additionally, ROS activate proteins, including forkhead box protein O (FOXO) transcription factors that regulate anti-oxidant enzyme expression and activate DNA repair pathways [10], [11]. Besides ROS, cellular metabolism also produces various other reactive chemical species, with a tendency to damage DNA such as alkylating agents and reactive nitrogen species. Recent studies showed the importance of FOXO-mediated oxidative stress defence for osteoblast function and skeletal homeostasis [12], [13].

Cells contain several DNA repair pathways. The global genome nucleotide excision repair (GG-NER) pathway removes helix-distorting damage anywhere in the genome, thus mainly preventing mutagenesis and consequently carcinogenesis. Transcription coupled repair (TCR) eliminates lesions in the transcribed strand of active genes when transcription is arrested by DNA damage, to allow rapid resumption of the vital transcription process. This repair system mainly promotes cell survival and prevents DNA damage induced cell death and senescence [14], [15]. Both repair processes share a number of components, including the 10-subunit transcription factor II H (TFIIH) complex [16]. TFIIH opens up the DNA helix using the helicases xeroderma pigmentosum complementation group B (XPB) and D (XPD) after damage detection in global genome as well as transcription-coupled repair [17]. This allows damage verification and excision by dual incision of the damaged strand at some distance from the lesion, followed by gap-filling DNA synthesis and final ligation to the pre-existing strand [18].

Mutations in the NER pathway helicase XPD, cause -among several other inherited syndromes- a disease called Trichothiodystrophy (TTD) which is a rare, autosomal recessive DNA repair disorder presenting a wide range of characteristic features like photosensitivity, brittle hair and nails, impaired intelligence, short stature, infertility and a severely reduced lifespan. Additionally, skeletal abnormalities have been described [19]. An XPD mouse model was generated, using murine homologue *Ercc2*, which precisely mimics a causative point mutation in the essential *Xpd* gene of a TTD patient, causing a single amino acid substitution (R^722^W) in the ERCC2 (XPD) helicase. Although mice were significantly lighter and smaller, radiographs of 2- to 4- month old TTD mice did not show any skeletal abnormalities, but those of 14 month old mice displayed kyphosis and a reduction in radiodensity of the long bones, both hallmarks of human aging [20]. In addition, TTD mice in a C57BL/6J background, have strikingly similar symptoms and show several premature aging-like features [21].

We used the TTD mouse model, which lacks one of the basic defense mechanisms against DNA damage and undergoes accelerated aging, to study the impact of DNA-repair in maintaining bone metabolism with aging. Previous studies using TTD mice have shown that early TTD development progresses normally when compared to wild type mice. Clear changes between the two groups start to develop after 26 weeks of age when TTD mice start to lag behind in gaining body weight and develop their characteristic features [22]. Early bone development was not affected and no changes in bone length were measured in male or female bones at any of the time points studied (data not shown). In this study we investigated bone architecture, gene expression and mesenchymal and hematopoietic stem cell differentiation potential.

## Results

### Bone Architecture

**Figure 1:**
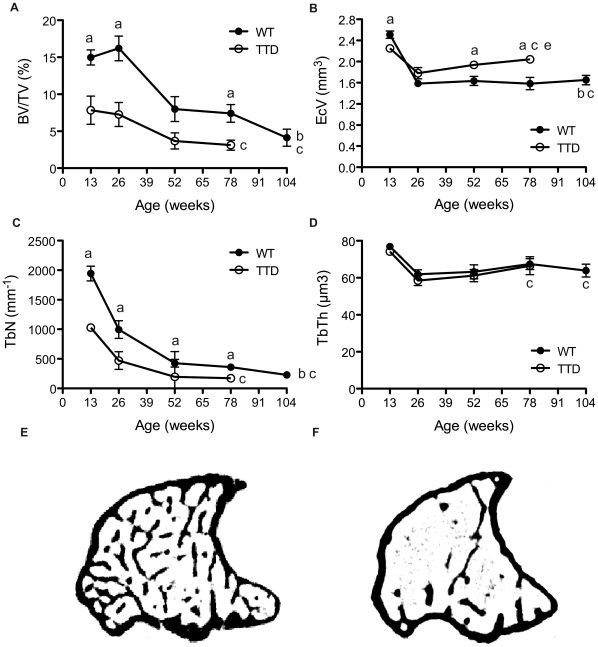
Age-related trabecular bone loss in male TTD mice. Trabecular bone parameters (A-D) were studied in aging wild type (solid) and TTD (open) tibial metaphyses. (A) Trabecular bone volume fraction (BV/TV), (B) Endocortical volume (Ec.V.), (C) Trabecular number (Tb.N.), (D) Trabecular thickness (Tb.Th.). 100 cross-sections were measured in the metaphysis of TTD and wild type mice. Representative cross-sections of 26 week old, male, wild type (E) and TTD mice (F) depict the loss of trabecular bone, the decrease in trabecular number and increase in endocortical volume. Statistics: a= student t-test; p<0.05 wild type vs. TTD, b−d = 2 way ANOVA; p<0.05. b= significant difference between wild type and TTD with aging, c= signifcant difference with aging within a genotype, d= a significant interaction between age and genotype. Error bars represent SEM.

In order to analyze bone dynamics in wild type and TTD mice throughout life we analyzed trabecular bone parameters in the metaphysis by µCT analysis. The trabecular bone volume fraction (BV/TV) gradually but significantly decreased over time in wild type mice, dropping 75% between 13 and 104 weeks of age. Albeit that at 13 weeks the BV/TV in TTD mice was already half of that in wild type mice, BV/TV decreased as well further throughout life, losing about 50% between 26 and 78 weeks of age (Fig. 1A). At all time points measured, BV/TV was significantly lower between wild type and TTD mice, with the largest difference observed at the first point measured at 26 weeks of age. No significant interaction between genotype and age was observed. The endocortical volume (Ec.V.), also known as the total volume contained by the endosteal surface of the cortical bone, was significantly lower in 13 weeks old TTD mice compared to wild type mice of the same age. Ec.V. decreased between 13 and 26 weeks in both wild type and TTD mice after which it stabilized in wild type mice. Ec.V. increased again In TTD mice, ending up significantly higher at 52 and 78 weeks of age compared to wild type mice (Fig. 1B). For Ec.V. there was a significant interaction between genotype and age, which means that age is a determinant of the difference between wild type and TTD mice. Trabecular number (Tb.N.) which decreased in wild types throughout life, is significantly lower in TTD mice, with the biggest difference observed at 13 weeks when TTD mice have about half the number of trabecules found in wild type mice of the same age (Fig. 1C). Trabecular thickness (Tb.Th.) however, was the same for both genotypes and did not change throughout life (Fig. 1D). In summary, TTD mice (Fig. 1F) displayed a reduction in trabecular bone compared to age-matched wild type mice (Fig. 1E) as shown in the sample cross-sections in Figure 1E and F. In addition, both wild type and TTD exhibited age-related decline.

**Figure 2:**
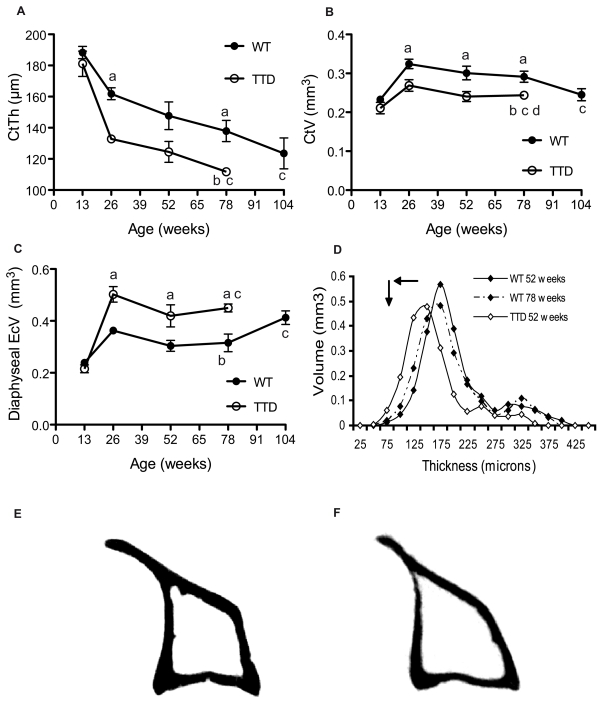
Age-related cortical bone loss in male TTD mice. Cortical bone parameters (A-D) were studied in aging wild type (solid) and TTD (open) tibial diaphyses.(A) Cortical thickness (Ct.Th.), (B) cortical volume (Ct.V.), (C) endocortical volume (Ec.V.), (D) 3D thickness distribution. Figures E and F depict the significant decrease in cortical thickness when comparing 26 week old wild type (E) and TTD (F) mice. Statistics: a= student t-test; p<0.05 wild type vs. TTD, b−d = 2 way ANOVA; p<0.05. b= significant difference between wild type and TTD with aging, c= signifcant difference with aging within a genotype, d= a significant interaction between age and genotype. Error bars represent SEM.

Cortical thickness (Ct.Th.) significantly decreased throughout life in wild type mice, losing approximately 20% between 13 and 104 weeks. The same gradual and significant decline was observed in TTD mice, which had thinner cortices compared to wild type mice at all time points measured, significantly so at 26 and 78 weeks (Fig. 2A). Cortical volume (Ct.V.) increased between 13 and 26 weeks after which it remained initially relatively stable in wild type mice, but rapidly and significantly dropped between 78 and 104 weeks of age reaching a level that is already obtained in TTD mice at 52 weeks. In TTD mice, Ct.V. also increased in the first time period, but less than in wild type mice, after which it stabilized throughout life but was significantly lower at all time points compared to wild type mice of the same age (Fig. 2B). Statistical analysis showed a significant interaction between age and genotype, which translated into a significant decrease in Ct.V. over time in TTD mice compared to wild type mice. Endocorital volume (Ec.V) followed a similar pattern in time as Ct.V (Fig. 2C). After the initial increase TTD mice have a relatively stable but significantly increased endocortical volume compared to wild type mice throughout their entire life. Ec.V. is stable up to 78 weeks of age in wild type mice after which it rapidly and significantly increases, reaching TTD levels (Fig. 2C). Analysis of the 3D thickness distribution within the diaphysis demonstrated a shift towards thinner bone fragments as well as a drop in the number of fragments when comparing young and old wild type as well as TTD mice. This is depicted for wild type mice in Figure 2D. Importantly, an accelerated age-related shift towards thinner fragments was observed in TTD mice when compared to wild type mice (Fig. 2D). In general, at the age of 6 months, TTD mice (Fig. 2F) already have much thinner cortices and less cortical bone compared to age-matched wild type mice (Fig. 2E).

Data of histomorphometric analyses on 13 and 78 week old wild type and TTD tibiae are shown in Table 1. None of the eleven studied histomorphometric parameters were significantly different at 13 weeks (Table 1). In contrast in 78 week old mice, several parameters were significantly lower in TTD mice, including Tb.N, Tb.Th. and BV/TV, supporting the µCT findings, while trabecular spacing (Tb.Sp) was significantly higher in TTD mice. For most parameters a significant interaction between age and the TTD genotype was shown (Table 1).

**Table 1:** Histomorphometric analyses.

	WT 13w (average ± SD)	TTD 13w (average ± SD)	p-value	WT 78w (average ± SD)	TTD 78w (average ± SD)	p-value	Interaction (p - value)
BV/TV (%)	4.06 ± 0.45	5.66 ± 0.84	0.19	7.47 ± 2.34	0.33 ± 0.10	0.05 *	0.003*
OV/BV (%)	1.02 ± 0.34	1.43 ± 0.68	0.65	0.43 ± 0.26	0.00 ± 0.00	0.07	0.311
OS/BS (%)	10.11 ± 1.62	20.35 ± 6.27	0.21	6.54 ± 3.51	0.00 ± 0.00	0.10	0.034*
ObS/BS (%)	10.48 ± 1.53	19.69 ± 5.56	0.20	6.19 ± 3.20	0.17 ± 0.15	0.05 *	0.022*
OcS/BS (%)	1.97 ± 0.26	2.74 ± 0.80	0.44	1.19 ± 0.23	0.00 ± 0.00	0.27	0.102
Tb.Th. (µm)	17.68 ± 1.14	22.11 ± 1.33	0.06	25.20 ± 3.71	10.94 ± 2.61	0.05 *	0.002*
O.Th. (µm)	0.77 ± 0.06	0.79 ± 0.19	0.93	0.62 ± 0.19	0.00 ± 0.00	0.07	0.032*
NOb/BPm (mm-1)	9.86 ± 1.32	18.10 ± 5.08	0.21	6.70 ± 2.78	2.84 ± 2.54	0.18	0.052
NOc/BPm (mm-1)	1.03 ± 0.14	1.5 ± 0.45	0.40	0.71 ± 0.14	0.00 ± 0.00	0.43	0.096
Tb.Sp. (mm)	431.84 ± 42.48	392.56 ± 46.68	0.60	441.28 ± 126.56	4202 ± 989.98	0.03 *	0.001*
Tb.N. (1/mm)	2.29 ± 0.2	2.52 ± 0.28	0.57	2.68 ± 0.57	0.27 ± 0.04	0.02 *	0.002*

Histomorphometric parameters were measured in 13 and 78 week old wild type and TTD tibiae (N=5). T-tests were performed at each time point, p-values are depicted in the table in the columns “p-value". Interaction between the age and phenotype of the mice was determined by performing 2-way ANOVA’s, p-values are depicted in the table in the column “Interaction". * = significant difference between wild type and TTD bones, p<0.05.

### Bone Strength

**Figure 3:**
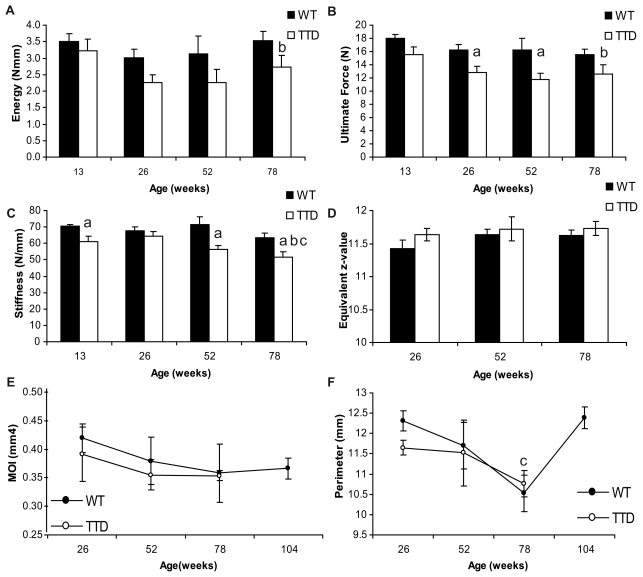
Decreased bone strength in TTD mice. Bone strength, mineralization, perimeter, MOI and bone formation rates in long bones of aging wild type (solid bars or symbols) and TTD males (open bars or symbols). (A) Energy to failure (B) Ultimate load (C) Bone stiffness, (D) bone mineralization, (C) MOI and (D) perimeter (Pm.). Statistics: a= student t-test; p<0.05 wild type vs. TTD, b−d = 2 way ANOVA; p<0.05. b= significant difference between wild type and TTD with aging, c= signifcant difference with aging within a genotype, d= a significant interaction between age and genotype. Error bars represent SEM.

Structural changes in bone architecture, like differences in cortices and alterations in matrix deposition can lead to changes in bone strength. To address this, we performed 3-point bending tests on wild type and TTD femurs. We measured three parameters; energy to failure, ultimate load and stiffness[23]. The amount of energy needed to break the bone – energy to failure – is with aging significantly lower in TTD that in wild type, but there was no significant interaction between genotype and age (Fig. 3A).

Wild type bones showed a gradual decrease in ulitmate force, reflecting general integrity of the bone structure, dropping from 18 N to 15.5 N over a period of 65 weeks (Fig. 3B). Ultimate force was similar in wild type and TTD mices at 13 weeks of age, consistent with the idea that initial bone development in TTD is not significantly affected. At 26 weeks, however, a drop to about 12.5 N was observed, which is in line with the differences found with other parameters at this age between TTD and wild type males. Bone stiffness was significantly decreased from 13 weeks onwards, with the exception of 26 week old bones (Fig. 3C). Quantitative backscatter scanning electron microscopic analysis of wild type and TTD femurs displayed no significant differences in mineralization of the cortex between genotypes or with aging (Fig. 3D). Two µCT parameters related to bone strength, moment of inertia (MOI) and perimeter (Pm.), did not show significant differences between wild type and TTD mice throughout life and followed similar patterns (Fig. 3E and Fig. 3F). MOI decreases gradually during life in both wild type and TTD mice as expected, since older bones usually become more fragile. Although the difference is not significant, TTD mice have consistently lower MOI values compared to wild type mice up to 78 weeks. In wild type mice in which an additional time point of 2 years was available, we observed a significant increase in perimeter after 78 weeks.

### Periosteal Apposition

**Figure 4:**
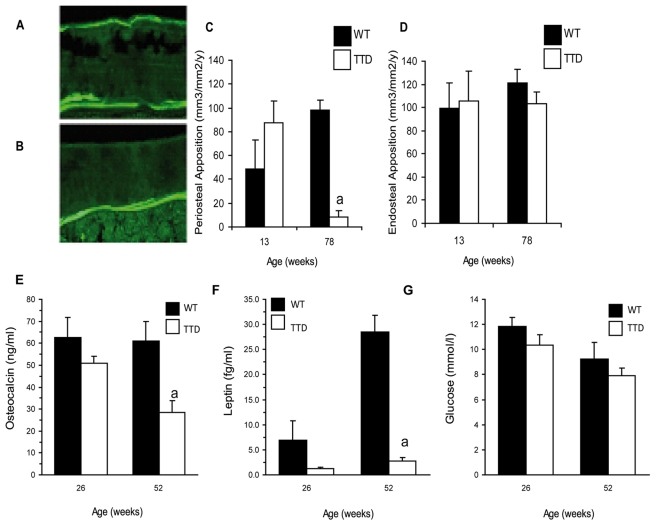
Periosteal apposition, bone formation and hormone levels. TTD mice lack periosteal apposition at 78 weeks whereas endosteal apposition is not affected. Calcein labelling at sites of periosteal apposition in 78-week-old (A) wild type and (B) TTD mice. Quantitative analyses of (C) periosteal and (D) endosteal apposition at 13 and 78 weeks. Serum measurements of bone formation markers and hormones; (E) osteocalcin, (F) leptin, (G) glucose at 26 and 52 weeks. Statistics: student t-test a = p<0.05 wild type vs. TTD. Error bars represent SEM.

An increase in bone perimeter at an older age is a distinctive feature of periosteal apposition, which generally takes place in aging bones in order to maintain bone strength by depositing new bone on the outside (periosteal) of the cortex in order to compensate for the intrinsic bone loss on the inside (endosteal). To address this further we measured periosteal bone formation rates using calcein labeling in wild type and TTD mice (Fig. 4A and Fig. 4B). Periosteal apposition was significantly reduced in old but not in young TTD mice (Fig. 4C). Endosteal bone formation was stable and unaffected throughout life (Fig. 4D).

Serum osteocalcin measurements demonstrated that at 52 weeks but not yet at 13 weeks of age TTD mice a have significant lower level of this bone formation parameter (Figure 4E). A characteristic phenotype of TTD mice is that they lack development of adipose tissue with aging [20]. This is reflected by the significant difference we measured in the adipocyte-derived cytokine leptin (Figure 4F). Irrespective of this strong difference the TTD were able to control their serum glucose level and maintain similar levels as the wild type mice (Figure 4G).

### Gene Expression

**Figure 5:**
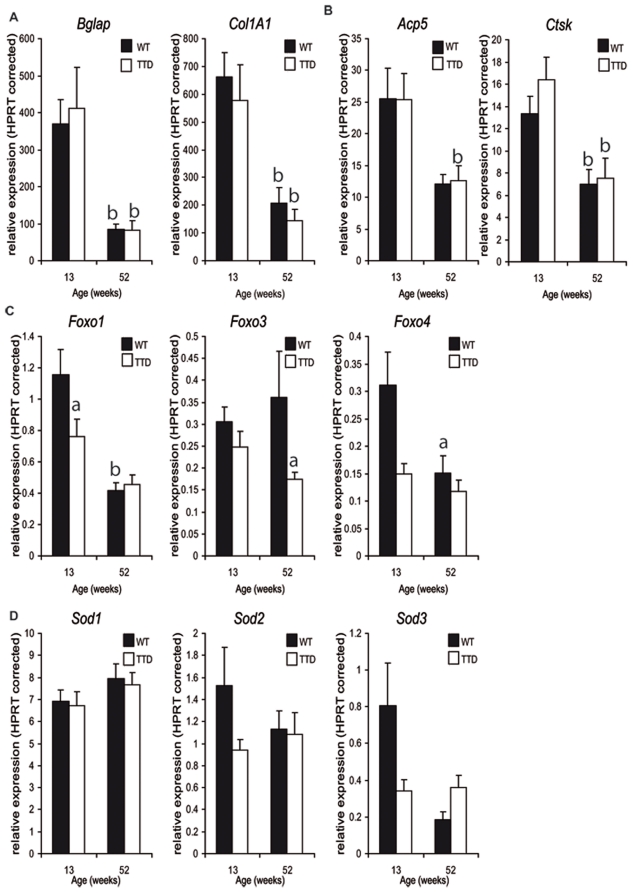
Regulation of gene expression levels in tibiae. Expression levels measured in RNA extracted from tibiae of 13 and 52 week old wild type and TTD mice; (A) osteoblast marker genes *Bglap* and *Col1A1*. (B) Osteoclast marker genes *Acp5* and *Ctsk*, (C) stress regulated genes *Foxo1, Foxo3* and *Foxo4*, (D) antioxidant enzyme scavengers *Sod1, Sod2* and *Sod3*. Statistics: student t-test a = p<0.05 wild type vs. TTD, b = p<0.05 time point x vs. time point 13 weeks within one genotype.

In order to gain more insight into the effects of a mutated *Ercc2 (Xpd)* gene on transcription in general and bone formation and remodeling specifically, we isolated RNA from tibiae and measured the expression levels of a number of osteoblast and osteoclast specific genes. Osteocalcin (*Bglap*) and collagen (*Col1A1*) were expressed at similar levels in wild type and TTD tibiae, both at 13 and 52 weeks. However, from 13 to 52 weeks of age, expression levels significantly decreased (Figs. 5A and B). Two well known osteoclast markers tartrate-resistant acid phosphatase (TRAP/ACP5) and cathepsin K (CTSK) were expressed at equal levels in wild type and TTD tibiae at both ages analyzed (Figs 5C and D). Similar as for the osteoblast markers, both Trap and Ctsk expression significantly decreased from 13 to 52 weeks of age.

Recently, the importance of the FOXO-mediated oxidative stress defence for osteoblast function and skeletal homeostasis has started to become clear [12], [13]. We studied the expression patterns of three Foxo genes as well as those of the three superoxide scavengers, *Sod1*, *Sod2* and *Sod3*. *Foxo1* was significantly lower at 13 weeks in TTD mice, whereas *Foxo3* and *Foxo4* were expressed at similar levels as in wild type tibiae. In 52 week old tibiae however, *Foxo3* and *Foxo4* expression was significantly decreased in TTD mice whereas *Foxo1* expression was similar in both genotypes (Fig. 5C). Both *Foxo1* and *Foxo4*, but not *Foxo3*, levels dropped when comparing 13 weeks old mice to 52 weeks old mice (Figs. 5C-E). All 3 Sod genes were expressed at similar levels in wild type and TTD mice throughout life. *Sod3* expression decreased from 13 to 52 weeks of age, whereas *Sod1* and *Sod2* expression remained at the same level in older mice (Fig. 5D). Next we analyzed the expression of pro- and anti-apoptotic genes in wild type and TTD mice at various ages. No significant difference in expression was observed at 13 and 52 weeks indicating that the observed skeletal phenotype in TTD mice is independent of apoptosis. At 104 weeks of age no consistent expression was observed supporting increased or decreased apoptosis. Pro-apoptotic genes were either higher (*Casp7* and *Casp9*) or lower (*Casp3* and *Casp6*) expressed in TTD tibiae (Figure S1).

### Bone Marrow Differentiation Potential

Besides a decrease in bone strength and a lack of periosteal apposition, older TTD mice also have a lack of body fat [21], [22], [24]. This could possibly be due to impairment in the number and/or differentiation capacity of mesenchymal stem cell (MSC), the common precursor of osteoblasts and adipocytes. In order to study this we isolated bone marrow containing MSCs and osteogenic and adipogenic progenitors from 26, 42 and 78 week old wild type and TTD mice and analyzed differentiation into osteoblasts and adipocytes in *ex vivo* cultures. In addition, we examined the osteoclastogenesis in these bone marrow samples.

**Figure 6:**
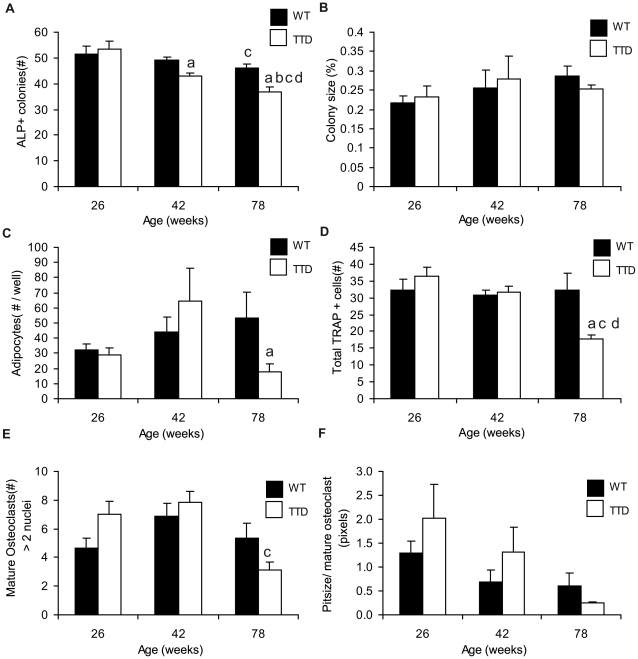
Mesenchymal and hematopoietic stem cell differentiation. Mesenchymal and hematopoietic stem cell differentiation in aging wild type (solid bars) and TTD males (open bars). (A) Average number of ALP^+^ colonies in ex vivo bone marrow cultures after 14 days of culture. (B) Average colony size at day 14 of culture. (C) The average number of adipocytes per well. (D) Average number of TRAP+ positive cells per picture. (E) Average number of mature osteoclasts per picture. (F) Average percentage resorption surface per osteoclast pit per mature osteoclast. Statistics: a= student t-test; p<0.05 wild type vs. TTD, b−d = 2 way ANOVA; p<0.05. b= significant difference between wild type and TTD with aging, c= signifcant difference with aging within a genotype, d= a significant interaction between age and genotype. Error bars represent SEM.

The number of alkaline phosphatase (ALP) positive colonies formed in TTD mice showed a significant age-dependent decrease and was at 42 and 78 weeks significantly lower than in wild type mice (Fig. 6A). No significant changes in colony size were measured with aging in wild type and TTD cells, and no significant differences between wild type and TTD cultures were observed indicating that at least *in vitro* proliferative potential was unaffected by aging and the TTD repair defect (Fig. 6B). Both wild type and TTD colonies mineralized to the same extent implicating unaffected mineralization in TTD mice (data not shown). In contrast to the number of osteoblast colonies, we did not observe a significant decrease in adipocyte differentiation potential as assessed by the number of lipid-vesicle-forming adipocytes in *in vitro* cultures (Fig. 6C).

Osteoclastogenesis analyses showed that the total number of TRAP+ (ACP5+) osteoclasts found in these *in vitro* cultures was stable throughout life in wild type mice up to the age of 78 weeks but significantly decreased in TTD mice (Fig. 6D). When focusing on multinucleated osteoclasts (>2 nuclei), no significant differences were observed between wild type and TTD mice although TTD cultures showed a significant trend towards a decline in multinucleated osteoclasts with aging (Fig. 6E). Additionally, osteoclasts were cultured on bone slices to analyze osteoclast resorption. Only mature osteoclasts are capable of resorbing bone, so we calculated the average size of a pit formed by a multinucleated osteoclast (Fig. 6F). For both wild type and TTD mice we observed a trend towards a decrease in resorption with aging, both of which did not reach significance (Fig. 6F). No significant differences were observed between wild type and TTD mice although at both 26 and 42 weeks, the resorbed area seemed about twice the size in TTD mice while at 78 weeks it was smaller (Fig. 6F).

## Discussion

The presented data reveal the significance of proper DNA repair and limiting DNA damage for bone metabolism and maintaining bone strength with aging. The TTD mice with impaired DNA repair display loss of bone structure and strength. Mice deficient in DNA repair are partially unprotected against DNA damage induced by ROS and other types of endogenous agents as has been shown in previous studies [25] and thereby the current study supports the concept of DNA damage-induced skeletal aging [1].

Our findings in aging wild type mice are in line with those presented in other studies that investigated male mice [4], [5]. Age-related bone loss in humans has been stipulated to be mostly restricted to cortical bone [6]. We do, however, see a clear decrease in trabecular bone volume (BV/TV) and trabecular number along side the decrease in cortical thickness and cortical volume that was expected. This suggests that not only cortical but also trabecular bone is subjected to age-related bone loss. These data correspond with those found in other studies [5]. Analyses of the 13 weeks old mice demonstrated a temporal difference in skeletal changes with age between trabecular and cortical bone. µCT analyses demonstrated already significant differences at trabecular bone sites between wild type and TTD mice while at cortical bone sites no differences were found. On basis of the trabecular data we cannot exclude that in male TTD mice the observed bone phenotype is partially the result of a developmental disturbance. Nevertheless, viewing our current histomorphometric analyses and the cortical data of 13 weeks old mice, we propose that the skeletal phenotype in male TTD mice is predominantly an age-related phenomenon instead of a developmental disorder.

We postulate that up to a certain age, somewhere between 13 and 26 weeks, the still functional DNA repair mechanisms in the TTD mice are able to limit accumulation of DNA damage. This limits cellular malfunctioning and premature cell death, and prevents skeletal alterations. However, this fails with progressing age, leading to premature skeletal aging. In addition, the fact that clear changes between TTD and wilt type only start to develop after 26 weeks of age is inherent to the fact that there is an important temporal component in the process from altered bone cell function to altered bone turnover and eventual overt skeletal structural changes.

The large difference in trabecular bone volume and cortical thickness in 26 weeks old male TTD mice may partially explain the reduction in bone strength that is already apparent at that age in TTD mice compared to wild type mice as we do not detect any significant changes in bone perimeter. In addition, decreased stiffness has previously been correlated with a loss in ultimate strength and an increased fracture risk [26]. In view of these data, we would also expect a decrease in MOI in these young mice, which appears not to be the case in our µCT analysis. However, MOI in these analyses is only a computational proxy for bone strength and does not take into account bone composition, making three point bending tests the more reliable measure. An interesting observation with respect to maintaining bone strength with aging is the profound lack of periosteal apposition in TTD mice. Bone is added to the outside of the cortex in order to compensate loss at the endocortical side and to maintain bone strength as long as possible [27], [28]. Disturbances in periosteal apposition and lack to compensate endocortical bone loss will lead to decreased bone strength and increased fracture risk. Involvement of estradiol, with opposite roles for estrogen receptors α and ß, testosterone and mechanical loading in the control of periosteal apposition have been reported [3]. However, the precise mechanisms underlying these age-related changes in bone and periosteal apposition are still poorly understood. Even though the current data do not yet provide a mechanism behind the lack of periosteal apposition, TTD mice might prove an excellent model to further unravel this important skeletal process with aging.

We did not observe an effect on endosteal apposition, whereas periosteal apposition id severely reduced. This might be explained by the segmental nature of the TTD phenotype, which has been previously observed. Where several organs and tissues in TTD mice are exposed to extremely accelerated aging, others are not affected at all [21], [22], [29]. These segmental effects might be due to differences between cell types, differences in timing and differences in intracellular metabolic stress levels. In the case of periosteal and endosteal apposition, timing might be the most important factor. Endosteal apposition is an important process early on in life, when redundant DNA repair mechanisms may still be able to limit the DNA damage. However, with aging periosteal osteoblast activity is the most important process to compensate for the age-related increased endosteal bone loss. This allows greater impact of DNA damage on periosteal than on endosteal apposition with aging.

The observed reduction in bone volume and perimeter throughout life in wild type and TTD mice will lead to a loss of bone strength. Up to 78 weeks of age there is a small decrease in perimeter which is in line with previous reports on cross-sectional area up to 20 months of age in male wild type mice [4]. Apparently, an increase in perimeter is not important to maintain bone strength up to this point in life. However, after 78 weeks wild type mice demonstrate the known age-related increase in perimeter due to periosteal apposition. Unfortunately, perimeter data on TTD mice over 78 weeks of age could not be obtained. Nonetheless, as TTD mice show a strong decrease in periosteal apposition at 78 weeks, it is unlikely that they would have shown an increase in perimeter as seen in wild type mice. This implicates that with progressing age and improper DNA repair a further and stronger decrease in bone strength will occur. The observed age-related difference in serum osteocalcin is supportive for a decreased periosteal bone formation.

Since TTD mice carry a specific defect in TCR combined with a partial GG-NER deficiency they are predicted to suffer more from the consequences of cellular DNA damage notably an increase in senescent or apoptotic cells. Indeed it has previously been observed that TTD mice even have a decreased spontaneous tumor formation rate. However, overprotection from cancer likely caused by enhanced death of cells with DNA damage may at the same time trigger accelerated aging [15], [22], [30], [31]. In order to get a first tentative look at how TTD cells might be affected by accumulating damage, we studied the mRNA expression of a number of oxidative stress related genes. FOXO proteins play an important role in the cell’s defense against oxidative stress and accumulating damage. In addition, FOXO1 can control bone mass. Osteoblast lineage specific deletion of *Foxo1* leads to a decrease in osteoblast number, bone formation rate and bone volume [13]. Interestingly we already observed a significant decrease in *Foxo1* expression in 13 weeks old TTD mice. This indicates that already at this young age, one of the fundamental mechanisms that protect the cells against stress is decreased and potentially contributing to decrease in bone structure in TTD mice. Further support for a role of the FOXO proteins is provided by the reduced expression of *Foxo3* and *Foxo4* at 52 weeks in TTD mice. Our analysis current analyses didn’t reveal a major difference in the expression of three *Sod* genes. To address these antioxidant mechanisms, additional detailed analyses at protein and functional level are needed but these were beyond the scope of our current study. The analysis of apoptosis genes in whole tibae suggest that the skeletal phenotype observed in TTD mice is independent of apoptosis. Up to 52 weeks no significant differences between wild type and TTD mice were observed while at 104 weeks pro-apoptotic genes were both down- and up-regulated in TTD mice. As these analyses are based on expression in whole tibae differences in apoptotic gene expression in specific subsets of cells (e.g. osteoblasts, osteoclasts, bone marrow cells including hematopoietic en mesenchymal stem cells) might have been missed and will be part of future studies to unravel the role of apoptosis.

An increase in damage and apoptosis can lead to a decrease in the number of stem and progenitor cells and or change their differentiation potential. Bone marrow harbors both mesenchymal and hematopoietic stem cells and their differentiation is tightly regulated in order to maintain a balance between the number of osteoblasts and osteoclasts [32], [33], [34]. Furthermore, the balance between osteoblast and adipocyte formation within the mesenchymal lineage is known to be regulated and can be tipped either way by disrupting one of the differentiation pathways [35]. The age-related decrease in wild type osteoblast colony formation potential we describe is in line with previous data [36].

Our current ex vivo TTD bone marrow cultures, as well as the histomorphometric data, showed a reduction in the capacity to form bone nodules and a trend towards reduced osteoclast activity with age. Taking into account that systemic factors might even increase the effect of this significant reduction in osteoblasts and osteoclasts, this could cause a reduction in bone turnover capacity and thereby a reduced capacity to maintain healthy bone. It is tempting to conclude that this is part of the mechanism underlying the observed early onset of skeletal aging and decrease in bone strength, although the observed effects are too small to be the only contributing factor. In addition, earlier in life, at 26 and 42 weeks of age, the number of multinucleated osteoclasts and resorbed area are higher in TTD mice than in wild type mice, which may also contribute to the increase in bone loss and increased endocortical volume earlier in life. This effect on osteoclasts is supported by preliminary FACS analysis of wild type and TTD bone marrow (data not shown) showing a shift in HSC differentiation towards the myeloid lineages in TTD mice.

Overall, these effects on osteogenic and osteoblast precursors and on osteoclastogenesis in TTD mice may contribute to accelerated bone aging and loss of bone strength in case of compromised DNA repair and accumulation of DNA damage. Although the effects on osteoblast and osteoclast formation are relatively mild, they are present for a prolonged time, which might expand and accumulate their final impact on bone formation. Additionally *in vivo* systemic processes are most likely involved in causing the observed changes in bone formation. Hormone levels, cytokines and growth factors may change with age [37] and previous studies on TTD and other DNA repair deficient mice show that they play an important role in causing the accelerated aging phenotype observed in these mice. For example the growth hormone/insulin-like growth factor cascade is impaired in other murine DNA repair deficient mouse models (Csb/Xpa and Xpf/Ercc1 mice) and a significant downregulation of the IGF axis was measured in TTD livers, indicating that this might well be the case in TTD mice as well [25], [38], [39]. Downregulation of this axis will skew the cells towards survival instead of differentiation pathways. Another study using TTD mice has shown a disturbed PPAR target gene regulation, which can have wide ranging systemic effects. This might well explain the discrepancy between the results from our *ex vivo* adipocyte cultures and our *in vivo* observations on adipocyte tissue formation. PPAR target genes play an important role in regulating adipocyte differentiation and a disrepancy in their regulation might very well be the cause of the characteristic lack of fat deposition in the TTD mice [30].

The lack of adipose tissue in TTD mice disturbs their energy metabolism and has a profound effect on the leptin levels that are found in TTD serum. Leptin levels, which regulate appetite and subsequently food intake, are not significantly different in young wild type and TTD mice. At 52 weeks however, there is a profound and significant difference between wild type and TTD mice. Several studies have shown that leptin directly affects bone formation, reducing trabecular bone while stimulating cortical bone formation [40], [41]. Logically, a lack in leptin would cause a loss of cortical bone, which fits our observations and might be one of the underlying factors for the observed decrease in cortical bone and loss of periosteal apposition. However, this would not fit our current observations on trabecular bone implicating that, once again, additional factors are involved.

In conclusion, accumulation of DNA damage induces skeletal aging in DNA repair deficient male TTD mice. This effect does not seem be sex specific. Recent observations in female mice by our group also show accelerated skeletal aging and loss of bone strength [42]. Considering the effect of ROS in DNA damage and aging [1], [43], [44], [45]our study supports the importance of proper anti-oxidant defence mechanisms and optimal DNA repair for skeletal maintenance and maintaining bone.

## Materials and Methods

### Ethics Statement

As required by Dutch law, formal permission to generate and use genetically modified animals was obtained from the responsible local and national authorities. All animal studies were approved by the Dutch equivalent of the Institutional Animal Care and Use Committees, Erasmus MC Dier Ethische Commissie.

### Mice and Bones

All mice had a C57BL/6J background and all mice within one cohort were kept under equal conditions. For micro-computed tomography (µCT) analysis 8 wild type and TTD mice were sacrificed at various ages (13–104 weeks). Because of their reduced lifespan [22] TTD mice within the µCT cohort were not available for the last time point of wild type mice (104 weeks) and therefore were only analyzed till 78 weeks. Mice were killed by cervical dislocation. Femurs and tibiae were isolated and were either snap frozen in liquid nitrogen and stored at −80°C or fixed in Burkhardt, which was replaced by 70% ethanol after 3 days. For bone marrow isolation 10 wild type and TTD mice were sacrificed after which bones were isolated and used for bone marrow isolation or fixed in 10% formalin for further testing.

### Micro-computed Tomography

Fixed tibiae from wild type and TTD mice (n = 4−6) were scanned by µCT from proximal end to mid-diaphysis using the SkyScan 1072 microtomograph (SkyScan, Antwerp, Belgium) with a voxel size of 8.82 µm. Bone parameters were quantified using the following software packages, Nrecon, CT-analyze and Dataviewer and MatLab (The Mathworks. Natick, MA,USA) (http://www.skyscan.be/products/downloads.htm). For all mice, a metaphyseal (100 sections) and a diaphyseal (50 sections) area were selected for analysis starting respectively 30 or 400 sections below our offset landmark within the epiphyseal growth plate. We determined the following parameters; trabecular bone volume fraction (BV/TV), metaphyseal endocortical volume (metaphyseal Ec.V.), trabecular number (Tb.N), trabecular thickness (Tb.Th.), cortical thickness (Ct.Th.), cortical volume, (Ct.V) diaphyseal endocortical volume (diaphyseal Ec.V)., polar moment of inertia (MOI) and perimeter (Pm.) [46]. In addition, 3D thickness distribution was determined, using a larger standardized area located 3.7–6.9 mm from the proximal end of the bones [47].For additional analysis proximal tibiae from male wild type and TTD mice at 13 weeks of age were scanned in the Skyscan 1076 in vivo X-ray microtomograph (Skyscan, Kontich, Belgium) as previously described [48]. Two regions were analyzed using the CTAnalyser software package (Skyscan): cortical thickness (Ct.Th.) and cortical porosity were determined in 20 cross sections of the mid-diaphysis of the tibia, and bone volume fraction (BV/TV), trabecular thickness (Tb.Th.) and cortical thickness (Ct.Th.) were measured in 50 cross sections of the proximal metaphysis, starting directly underneath the growth plate."

### Histomorphometric Analysis

To assess dynamic histomorphometric indices, mice received intraperitoneal injections with calcein (10 µg/g body weight) 10 and 3 days prior to sacrifice. Tibiae and sections were processed and stained as described previously [49]. Parameters of static and dynamic histomorphometry were quantified on not decalcified proximal tibia sections. For each animal, the bone formation rate was determined by fluorochrome (calcein) measurements using two non-consecutive 12µm-sections per animal. Additional histomorphometric analysis was performed on 13 and 78 week old wild type and TTD bones as previously described [50].

### Mechanical Testing

Defrosted femurs were disposed of residual soft tissue and tested in a three-point bending assay using a Lloyd LRX mechanical test frame, constructed with 3 mm hemi-cylindrical supports with a 9 mm total span. The femurs were aligned such that the neutral axis was parallel to the sagittal plane. The lesser trochanter was used as a reference point and aligned with one of the two supports. All samples were preconditioned for 5 cycles to 2 N at a rate of 0.01 mm/s before testing to failure at a rate of 0.02 mm/s. We measured ultimate load (N), energy (Nmm) and stiffness (N/mm).

### Backscatter Scanning Electron Microscopy

Processing and quantitative backscatter scanning electron microscopic analyses were done as described previously [51].

From each image the histogram of equivalent Z-values was calculated, its mean value representing the mean mineralization of the bone tissue [24].

### Bone Marrow Isolation, Cell Culture and Cell Culture Staining

Bone marrow was collected immediately after the bones were obtained by spinning down the bone marrow into an Eppendorf tube at 5000rpm for 2 min. Erythrocytes were lysed using erylysis buffer (1.55M NH_4_Cl, 0.1M KHCO_3_, 1mM EDTA (10x)) and cells were washed and seeded at 1.000.000 cells/well (12 wells) for osteoblasts, 100.000 cells/well (96 wells) for osteoclasts and 750.000 cells/well, for adipocytes (24 wells). Osteogenic and osteoclastogenic cultures were performed as previously described [52]. Adipocytes were cultured on adipogenic (D-MEM (GIBCO, Paisley, UK) supplemented with P/S, amphotericin B (250 ng/ml, Sigma, Zwijndrecht, NL) and 15% heat-inactivated FCS (GIBCO) medium. Adipogenesis was induced after a 3-week expansion phase by adding insulin (0.1 µg/ml, Sigma), indomethacin (1 mM, Sigma) and dexamethasone (1*10^−7^M, Sigma) for two weeks. Osteoblast colony forming potential and mineralization potential were determined as previously described after respectively 14 and 21 days of culture [52]. Colonies were counted by eye, next plates were scanned and colony surface area was determined by computer analysis (Bioquant software, Nashville, TN, USA). Alizarin Red staining was extracted and quantified by absorptiometry at 405 nm on a plate reader (Victor^2^ 1420 multilabel counter, Wallac, Perkin Elmer, Waltham, MA, USA).

Osteoclast number and resorption capacity were determined as previously described [30] and analyzed using freely available Image J software (version 1.41;http://rsbweb.nih.gov/ij/). Adipocyte cultures were fixated O/N with 10% formalin, washed with 60% IPOH and stained with Oil-Red O solution (60–40 dilution of Oil-Red O stock solution (Clin-Tech limited, Guiltford, UK) in H_2_O) after which the total amount of adipocytes per well was determined.

### RNA Isolation and qPCR Analysis

After removing tibiae from the mice, we removed the bone marrow and immediately homogenized the tissue into Trizol using a Tissuelyser (Qiagen, Venlo, NL). RNA was isolated and purified using a Qiagen RNeasy kit (Qiagen). Total RNA amount was determined using a spectrophotometer (ND1000, Nanodrop, Thermo Scientific, Wilmington, DE, USA). For cDNA synthesis 1 µg of total RNA was reverse transcribed using a cDNA synthesis kit according to the manufacturer’s protocol (MBI Fermentas, Thermo Scientific, Wilmington, DE, USA). qPCR analysis was performed using a ABI 7500 Fats Real-Time PCR detection system (Applied Biosystems, Carlsbad, CA, USA). Reactions were performed in 25 µl volumes using a qPCR core kit (for assays using a probe) or a qPCR kit for SYBR green I (for assays using SYBR Green) (Eurogentec, Maastricht, NL). Primer and probe sets were designed using Primer Express software (version 2.0, Applied Biosystems).

### Statistics

We performed 2-way ANOVA statistical analysis on all datasets to determine any interactions between age and phenotypes, as well as additional unpaired t-tests with two-tailed P-values to determine significant difference at each time point (Graph Pad Software, San Diego, CA, USA, www.graphpad.com).

## Supporting Information

Figure S1 **Expression of apoptosis related genes in wild type and TTD tibiae at various ages.** Expression levels of anti- and pro-apoptotic genes were measured in RNA extracted from tibiae of 13, 52 and 104 week old wild type and TTD mice; (A-G) pro-apoptotic genes; Cycs, Bax, Casp3, Casp6, Casp7, Casp9 and Diablo (H-I) Anti-apoptotic genes; Bcl2 and Birc4. Statistics: student ttest a = p<0.05 wild type vs. TTD.
(TIF)
