# Supplementary figures and images for: Age-Related Skeletal Dynamics and Decrease in Bone Strength in DNA Repair Deficient Male Trichothiodystrophy Mice
Source: PLoS One. 2012 Apr 10;7(4):e35246. doi: 10.1371/journal.pone.0035246 (PMC3323647; doi:10.1371/journal.pone.0035246)

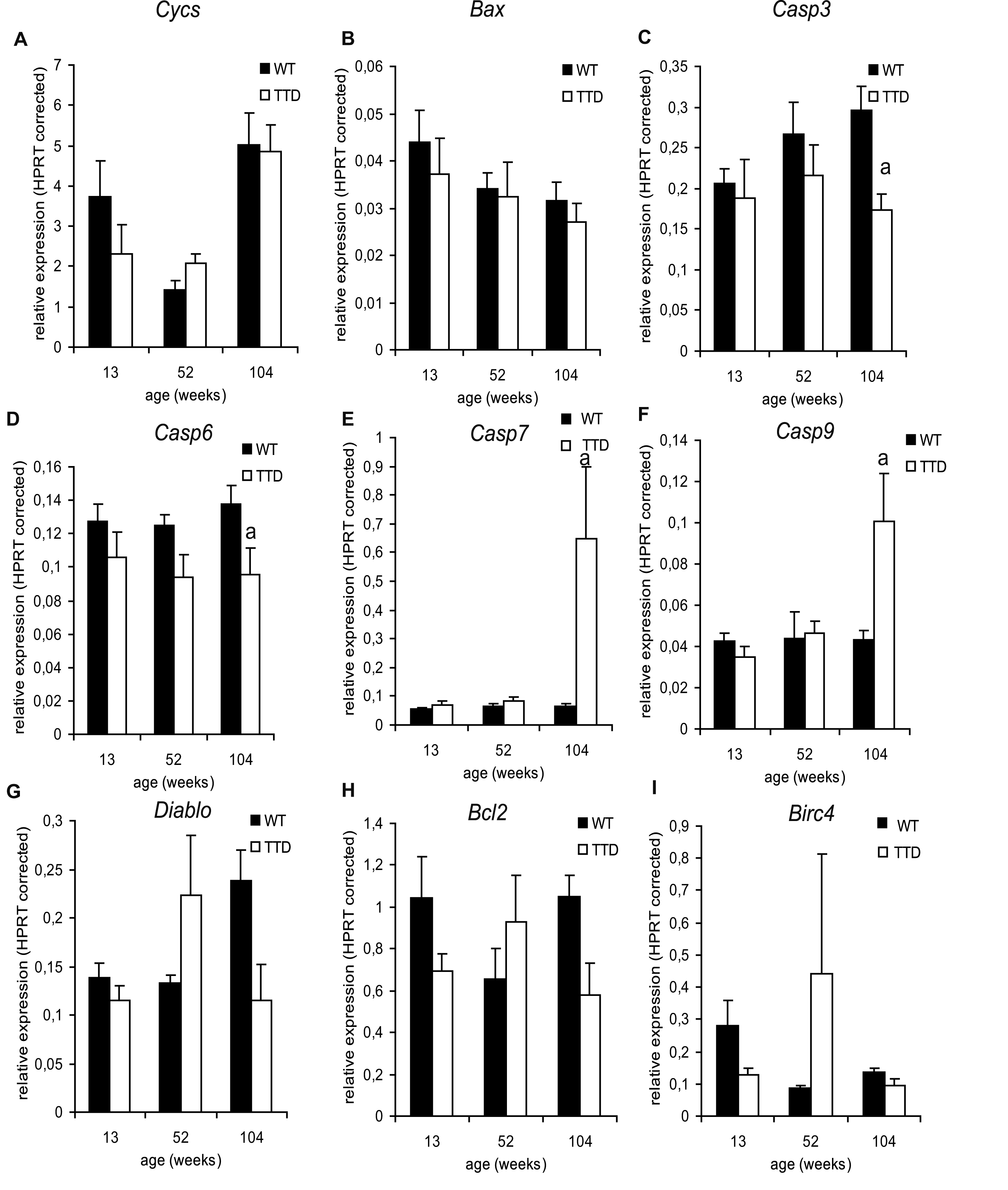

Supplement: Figure S1 — Expression of apoptosis related genes in wild type and TTD tibiae at various ages. Expression levels of anti- and pro-apoptotic genes were measured in RNA extracted from tibiae of 13, 52 and 104 week old wild type and TTD mice; (A-G) pro-apoptotic genes; Cycs, Bax, Casp3, Casp6, Casp7, Casp9 and Diablo (H-I) Anti-apoptotic genes; Bcl2 and Birc4. Statistics: student ttest a = p<0.05 wild type vs. TTD. (TIF) [file pone.0035246.s001.tif]
